# *Lacmellea oblongata* and Other Undervalued Amazonian Fruits as Functional, Antioxidant, and Antimicrobial Matrices

**DOI:** 10.3390/antiox14080924

**Published:** 2025-07-29

**Authors:** Elena Coyago-Cruz, Gabriela Méndez, Ruth Escobar-Quiñonez, Marco Cerna, Jorge Heredia-Moya

**Affiliations:** 1 Carrera de Ingeniería en Biotecnología, Universidad Politécnica Salesiana, Sede Quito, Campus El Girón, Av. 12 de Octubre N2422 y Wilson, Quito 170143, Ecuador; 2 Facultad de Ciencias de la Salud Eugenio Espejo, Centro de Investigación Biomédica (CENBIO), Universidad UTE, Quito 170527, Ecuador

**Keywords:** bioactive compounds, functional food, microextraction, ABTS, DPPH, well diffusion

## Abstract

The Amazon represents a key source of food biodiversity and is home to native fruits with high nutritional and functional potential, many of which remain largely unstudied. This research aimed to evaluate the presence of bioactive compounds, as well as the antioxidant and antimicrobial activity of *Miconia crenata*, *Grias neuberthii*, *Lacmellea oblongata*, *Pourouma cecprofiilia*, and *Annona edulis*. Physical and chemical parameters, mineral content (atomic absorption), vitamin C, organic acid, carotenoids, chlorophylls, and phenols (liquid chromatography), antioxidant activity (ABTS, DPPH), and antimicrobial activity (against *Candida albicans*, *Candida tropicalis*, *Escherichia coli*, *Staphylococcus aureus*, and *Streptococcus mutans*) were determined. High concentrations of calcium, syringic acid, and antioxidant activity were found in the fruits of *Miconia crenata*; malic and caffeic acids in *Grias neuberthii*; citric acid, naringenin, and antioxidant activity in *Lactuca oblongata*; potassium, chlorogenic acid, and ferulic acid in *Pourouma cecropiifolia*; and tartaric acid and gallic acid in *Annona edulis*. Additionally, low antimicrobial activity was observed in *M. crenata* against *E. coli* (2.7 mg/mL), *G. neuberthii* against *S. aureus* (10.3 mg/mL), and *L. oblongata* against *S. mutans* (10.4 mg/mL), *C. albicans* (20.8 mg/mL), and *C. tropicalis* (20.8 mg/mL). The results confirm that these Amazonian fruits are a relevant source of functional bioactive compounds, highlighting their potential for use in the food, pharmaceutical, and biotechnology sectors.

## 1. Introduction

In addition to meeting human nutritional needs, food can influence health and disease states, leading to the concept of nutraceuticals. These are substances that reduce the impact of chronic diseases on public health, particularly in contexts of population ageing and unhealthy lifestyles [1]. The increase in non-communicable diseases, including cardiovascular and obesity disorders, has reached worrying levels, necessitating dietary strategies based on functional foods rich in bioactive compounds derived from natural plant sources, marine organisms, and animals [2].

In this sense, consuming non-conventional fruits is a promising way to counteract the adverse effects of the Western diet, which is characterised by high consumption of ultra-processed products that are rich in fats and sugars. This diet is associated with low energy expenditure and a sedentary lifestyle. A balanced diet is essential for health at all stages of life, and consuming foods rich in natural antioxidant compounds can modulate metabolic pathways related to disease prevention. Thus, fruits are important dietary sources of fibre, essential vitamins, and minerals, as well as a variety of bioactive compounds, including phenolic compounds, carotenoids, and betalains [1,3].

The Amazon is one of the regions with the most remarkable plant diversity, harbouring species that have not been extensively explored and which have nutraceutical potential. The rediscovery of ancestral species has sparked growing interest due to their functional value and contribution to food and nutritional security [1,4]. Several studies have documented the presence of bioactive molecules in Amazonian fruits that have received little scientific attention—for example, a review of carotenoids in tropical fruits [2], analyses of bioactive compounds in Amazonian fruits [5], a study of non-traditional fruits such as tintiuk and non-traditional varieties of cocoa [4], and the characterisation of 51 non-conventional tropical fruits [6].

Most of these Amazonian fruits are grown using subsistence farming methods for self-consumption. Productivity is low due to the limited utilisation of technological innovations and the lack of a structured value chain. Nevertheless, international organisations and governments have promoted their consumption due to their proven health benefits [3,5]. The growing interest in these fruits is attributable to their antioxidant, anti-inflammatory, hypocholesterolemic, anti-obesity, and protective attributes against metabolic syndrome, which are ascribed to the existence of vitamins, carotenoids, and polyphenolic compounds [7,8].

Tropical fruits and their derivatives constitute an abundant reservoir of bioactive molecules possessing antimicrobial attributes that may confer advantages to human health. They contain high levels of flavonoids, anthocyanins, and carotenoids, which play a crucial role in their beneficial effects on health [9]. These secondary metabolites display a broad range of functional activities, such as mitigating oxidative stress, reducing inflammation, delaying aging processes, and offering protection against UV radiation. As a result, they are considered valuable for both the food sector and the cosmetic industry [3,10].

Various fruit extracts, including those from citrus fruits, berries, pomegranates, and non-traditional cocoa sources, have been shown to exhibit antimicrobial properties against pathogenic bacteria by disrupting cell membranes and suppressing key metabolic functions. Infectious diseases remain among the primary contributors to global mortality. Many of these infections are bacterial in origin and are conventionally treated with antibiotics. However, the increasing prevalence of antimicrobial resistance has driven the need to identify novel therapeutic options [4,11].

In this context, numerous studies have demonstrated that compounds extracted from plants exhibit antimicrobial or medicinal properties, establishing tropical fruits as a natural source of phytochemicals that can be used to prevent and control pathogens [4]. Compounds such as organic acids, including citric acid, malic acid, tartaric acid, propionic acid, acetic acid, and salicylic acid, as well as terpenes, flavonoids, and other non-flavonoid polyphenols, have been shown to exhibit significant antimicrobial activity [11]. Consequently, phenolic compounds have gained attention as potential antimicrobial agents against clinical pathogens. These secondary metabolites may interfere with key microbial growth processes, including the inhibition of bacterial growth, the suppression of biofilm development, and the alteration of vital metabolic pathways. The mechanisms of action are diverse and include disruption of cell wall synthesis, inhibition of DNA replication, and modulation of enzymes [12,13].

Phenolic compounds such as cinnamic, gallic, 4-hydroxybenzoic, *p*-coumaric, syringic, and vanillic acids, along with catechin, epicatechin, quercetin, and resveratrol, are among the most extensively examined for their antimicrobial efficacy. These compounds are effective against a variety of pathogenic microorganisms, including *Bacillus subtilis*, *B. cereus*, *Escherichia coli*, *Listeria monocytogenes*, *Pseudomonas putida*, *P. aeruginosa*, *Salmonella enterica*, and *Staphylococcus aureus* [14].

In this context, several Amazonian species have been documented as having traditional uses and phytochemical profiles with functional and therapeutic potential. Species of the *Miconia* genus have traditionally been employed in folk medicine for the management of ailments such as pain, throat infections, fever, and colds, and their depurative, diuretic, and sedative properties [15]. *Grias neuberthii* o sacchamangua, on the other hand, is a notable source of lipids, fibre, and protein, making it a promising candidate for nutritional and culinary applications [16,17]. *Pourouma cecropiifolia*, or caimaron, or uvilla, contains flavonoids and polyphenols with recognised antioxidant properties that contribute to health maintenance by neutralising free radicals and protecting against chronic diseases [18]. Phenolic compounds associated with high antioxidant capacity and potential health benefits have been identified in *Annona* species, including *A. edulis* [19]. However, no studies characterising the bioactive compounds present in *Lacmellea oblonga* have yet been reported, highlighting a knowledge gap and the need for research aimed at exploring their phytochemical and functional profile. This research sought to characterise bioactive compounds and evaluate the antioxidant and antimicrobial potential of neglected Amazonian fruits, offering evidence for their use as functional ingredients in food, medicine, and biotechnology.

## 2. Materials and Methods

### 2.1. Reagents and Standards

Analytical grade hydrochloric acid (CAS 7647-01-0) was purchased from Labscan (RCI Labscan, Dublin, Republic of Ireland). Meanwhile, analytical grade sodium hydroxide (CAS 1310-73-2), fluconazole (CAS 86386-73-4), acetone (CAS 67-64-1), DPPH (2,2-diphenyl-1-picrylhydrazine) (CAS 1898-66-4), formic acid (CAS 64-18-6), Folin–Ciocalteu (CAS 7732-18-5), potassium chloride (CAS 7447-40.7), potassium persulfate (CAS 7727-21-1), sodium acetate trihydrate (CAS 6131-90.4), nitric acid (CAS 7697-37-2), sodium carbonate (CAS 497-19-8), metaphosphoric acid (CAS 37267-86-0), and ABTS (2,2′-azino-bis-(3-ethylbenzothiazoline-6-sulfonic acid)) (CAS 30931-67-0) were obtained from Sigma-Merck (Darmstadt, Germany). Chromatographic grade acetonitrile (CAS 75-05-8), ethanol (CAS 64-17-5), and methanol (CAS 67-56-1) were supplied by Fisher Chemical (Fisher Scientific Inc., Madrid, Spain).

Analytical standards including chlorogenic acid (95.0%, CAS 327-97-9), chrysin (97.0%, CAS 480-40-0), ferulic acid (100.0%, CAS 1135-24-6), and gallic acid (100.0%, CAS 149-91-7), 2,5-dihydroxybenzoic acid (98.0%, CAS 490-79-9), 3-hydroxybenzoic acid (99.0%, CAS 99-06-3), kaempferol (97.0%, CAS 520-18-3), luteolin (98%, CAS 491-70-3), CAS 588-30-7), naringin (95.0%, CAS 10236-47-2), caffeic acid (98.0%, CAS 331-39-5), *p*-coumaric acid (98.0%, CAS 501-98-4), *p*-hydroxybenzoic acid (99.0%, CAS 99-06-3), quercetin (95.0%, CAS 849061-97-8), rutin (94.0%, CAS 153-18-4), *m*-coumaric acid (99.0%, shikimic acid (99.0%, CAS 138-59-0), syringic acid (95.0%, CAS 530-57-4), *o*-coumaric acid (97.0%, CAS 614-60-8), vanillic acid (97.0%, CAS 121-34-6), trolox (98%, CAS 53188-07-1), and β-carotene (93.0%, CAS 7235-40-7) were purchased from Sigma-Merck (Darmstadt, Germany). For mineral analysis, standard solutions of iron (CAS 7439-89-6), sodium (CAS 7440-23-5), magnesium (CAS 7439-95-4), potassium (CAS 7440-09-7), and calcium (CAS 7440-70-2) at a concentration of 1000 μg/mL were obtained from Accustandard (AccuStandard, Inc., New Haven, CT, USA).

In microbiological assays, culture media such as Mueller–Hinton agar (MHA), brain heart infusion (BHI), and Sabouraud dextrose agar (SDA) were sourced from BD Didcot (Fisher Scientific Inc., Madrid, Spain). Dextrose-yeast peptone broth (YPDB) was obtained from SRL (Sisco Research Laboratories Pvt. Ltd., Bombay, India), and streptomycin sulphate (CAS 3810-74-0) was acquired from Phytotech (PhytoTechnology Laboratories^®^, Lenexa, KS, USA). The bacterial strains used in this study included *Pseudomonas aeruginosa* (ATCC 9027), *Streptococcus mutans* (ATCC 25175), *Staphylococcus aureus* (ATCC 6538P), and *Escherichia coli* (ATCC 8739). The yeast strains were *Candida albicans* (ATCC 1031) and *Candida tropicalis* (ATCC 13803). All microbial strains were obtained from the American Type Culture Collection (ATCC) in Manassas, VA, USA. Ultrapure water used throughout the experiment was obtained using a NANOpure Diamond system (Barnstead Inc., Dubuque, IA, USA).

### 2.2. Physicochemical Analyses

**Figure 1:**
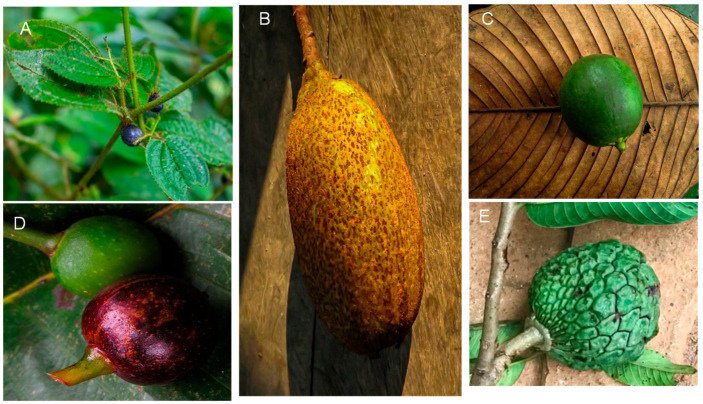
Photograph of fruit in study. Note: (**A**), *Miconia crenata* (Vahl) Michelang. (4759, Herbarium QUPS-Ecuador); (**B**), *Grias neuberthii* J.F.Macbr. (4758, Herbarium QUPS-Ecuador); (**C**), *Lacmellea oblongata* Markgr. (4753, Herbarium QUPS-Ecuador); (**D**), *Pourouma cecropiifolia* Mart (4750, Herbarium QUPS-Ecuador); (**E**), *Annona edulis* (Triana & Planch.) H.Rainer (4785, Herbarium QUPS-Ecuador).

Edible fruit samples were acquired from indigenous communities within the Ecuadorian Amazon region (Figure 1), specifically in the province of Pastaza (1° 44′21″ S, 77°29′1″ W). For taxonomic identification, the plant material was pressed and examined at the herbarium of the Salesian Polytechnic University in Quito, Ecuador.

To ensure sample homogeneity, ripe fruits were randomly collected from ten plants concentrated within a defined area of the study site, on the sunny side of each plant. The sample size varied depending on the size of the fruit: two kilograms of ripe fruit were collected for small fruits, such as *Miconia crenata*, and 50 ripe fruits were collected for larger fruits [20]. The samples were divided into two portions: one was used to determine physicochemical characteristics, while the skin was removed from the other and then freeze-dried to determine its bioactive compounds, antioxidant capacity, and antimicrobial activity. Before freeze-drying, the fruit pulp was frozen at −80 °C and subsequently dried using a Christ Alpha 1-4 LDplus freeze dryer (Martin Christ GmbH, Osterode am Harz, Germany). The resulting freeze-dried material was ground into a fine powder using a laboratory mill and stored in sealed glass containers under a nitrogen atmosphere at −20 °C until analysis.

Physicochemical characterisation of the fresh fruit involved determining its weight, longitudinal diameter, and equatorial diameter. The pH was measured using a SevenMulti S47 (Mettler Toledo, Columbus, OH, USA). Soluble solids were measured using a handheld refractometer and acid-base titration. Moisture content was measured by gravimetry at 100 °C in a Memmert Be 20 oven (Memmert GmbH + Co. KG, Barcelona, Spain). Ash content was determined by calcining the sample at 550 °C in a muffle furnace (Thermo Fisher Scientific, Waltham, MA, USA) [4].

#### Mineral Profile

A quantity of 40 mg of freeze-dried fruit powder was accurately weighed and transferred into Teflon digestion vessels compatible with a Speed-180 wave Xpert microwave digestion system (Berghof Products + Instruments GmbH, Eningen unter Achalm, Germany). Each sample was treated with 5 mL of 65% nitric acid and left to stand for 10 min to initiate the pre-digestion process. The vessel was then sealed according to the manufacturer’s specifications and subjected to microwave-assisted digestion. After cooling to room temperature (approximately 20 min), the digested samples were diluted to a final volume of 25 mL with Milli-Q water.

The concentrations of selected minerals (calcium (Ca), iron (Fe), sodium (Na), magnesium (Mg), and potassium (K)) were determined using a Varian SpectrAA-55 atomic absorption spectrophotometer (Varian Inc., Palo Alto, CA, USA). Calcium was quantified using a wavelength of 422.7 nm with a slit width of 0.5 nm and a mixture of acetylene and nitrous oxide. For the other minerals, a mixture of air and acetylene was used: iron at 372.0 nm with a slit width of 0.20 nm; sodium at 589.6 nm with a slit width of 0.5 nm; potassium at 404.4 nm with a slit width of 0.5 nm; and magnesium at 202.6 nm with a slit width of 1.0 nm. The standard solutions had a base concentration of 1000 ppm, from which specific dilutions were prepared in the following ranges: 0–5 ppm for calcium; 0–20 ppm for iron; 0–200 ppm for potassium; 0–10 ppm for magnesium; and 0–8 ppm for sodium. Mineral concentrations were expressed as milligrams per 100 g of dry weight (mg/100 g DW) [4].

### 2.3. Analysis of Bioactive Compounds

#### 2.3.1. Ascorbic Acid

Ascorbic acid was quantified using liquid chromatography [21]. For the quantification of vitamin C, *L*-ascorbic acid was used as the standard for analysis. A mass of 20 mg of lyophilised powder was suspended in 1.2 mL of 3% metaphosphoric acid and 200 µL of 0.2% homocysteine and homogenised using a VM-300 vortex mixer (Interbiolab Inc., Orlando, FL, USA). Subsequently, 600 mL of deionised water was added, and the solution was filtered before analysis. Chromatographic preparation was performed using an RRLC 1200 high-resolution liquid chromatograph (Agilent Scientific Instruments, Santa Clara, CA, USA) coupled with a DAD-UV-VIS detector and a ZORBAX Eclipse XDB80 AC C18 column (1.8 µm, 4.6 mm × 50 mm) (Agilent Scientific Instrument, Santa Clara, CA, USA). The mobile phase consisted of 90% 1.5% monobasic potassium phosphate dissolved in deionised water and 10% 1.8% n-cetyl-n,n,n-trimethylammonium bromide dissolved in HPLC grade methanol, delivered at a constant flow rate of 1 mL/min. The injection volume was 20 µL, and detection was carried out at 244 nm. Standard solutions of *L*-ascorbic acid were prepared at a concentration of 1 mg/mL, a dilution range of 0.16–1.00 mg/mL with five points, and with an R^2^ value greater than 0.99. Vitamin C concentration was expressed as milligrams per 100 g of dry weight (mg/100 g DW).

#### 2.3.2. Organic Acid Profile

Individual organic acids were quantified using liquid chromatography [21]. For extraction, 30 mg of the freeze-dried sample was extracted with 1.5 mL of 0.02 N sulfuric acid containing 0.05% metaphosphoric acid and 0.2% homocysteine. The mixture was homogenised using a VM-300 vortex mixer (Interbiolab Inc., Orlando, FL, USA), followed by sonication in an FS60 ultrasonic bath (Fisher Scientific Inc., Waltham, MA, USA) to enhance compound extraction. The resulting extract was filtered before chromatographic analysis. Quantification was performed using a 1200 Rapid Resolution Liquid Chromatography system (RRLC) (Agilent Scientific Instruments, Santa Clara, CA, USA) equipped with a diode array detector (DAD-UV-VIS) and a YMC-Triart C18 column (3 µm, 4.6 mm × 150 mm) (YMC Europe GmbH, Dinslaken, Germany). The mobile phase consisted of 0.027% sulfuric acid in ultrapure water, delivered at a constant flow rate of 1 mL/min. The injection volume was 20 µL, and detection was carried out at 210 nm. Standard solutions of tartaric acid, citric acid, and malic acid were prepared at a concentration of 1 mg/mL, a dilution range of 0.05–1.00 mg/mL with five points, and with an R^2^ greater than 0.99. Identification was performed by comparing the corresponding spectra and retention time. Results were expressed as milligrams per 100 g of dry weight (mg/100 g DW).

#### 2.3.3. Carotenoid Profile

Individual carotenoids were quantified using liquid chromatography [22]. Identification and quantification of individual carotenoids were carried out using certified reference standards of astaxanthin, α-carotene, β-carotene, β-cryptoxanthin, lycopene, lutein, trans-β-apo-8-carotenal, violaxanthin, and zeaxanthin. For extraction, 20 mg of freeze-dried powder samples was mixed with 250 µL of methanol, 500 µL of chloroform, and 250 µL of Milli-Q water. The mixture was homogenised using a VM-300 vortex mixer (Interbiolab Inc., Orlando, FL, USA), followed by sonication in an FS60 ultrasonic (Fisher Scientific Inc., Waltham, MA, USA). This extraction procedure was repeated until the solid residue was utterly devoid of colour, indicating the complete removal of carotenoids. The resulting coloured extract was evaporated to dryness under reduced pressure using a rotary vacuum evaporator at temperatures below 30 °C.

The dried extract was redissolved in 20 µL of ethyl acetate prior to analysis. Carotenoids were quantified using a 1200 Rapid Resolution Liquid Chromatograph (RRLC) system (Agilent Technologies, Mississauga, ON, Canada), equipped with a diode array detector (DAD-UV-VIS) and a YMC C30 column (3 µm, 4.6 × 150 mm) (YMC Europe GmbH, Dinslaken, Germany). The mobile phase flow rate of 1 mL/min, considered a gradient of acetonitrile HPLC grade (A), methanol HPLC grade, and ethyl acetate HPLC grade, was as follows: 85% A + 15% B, 0 min; 60% A and 20% B + 20% C, 5 min; 60% A + 20% B + 20% C, 7 min; 85% A + 15% B, 9 min; and 85% A + 15% B, 12 min. The injection volume was 20 µL, and detection was carried out at 250 nm for phytoene and phytofluene and 450 nm for α-carotene, β-carotene, β-cryptoxanthin, lycopene, lutein, and violaxanthin. Identification was performed by comparing the corresponding spectra. Quantitation was based on individual calibration curves with a concentration of 1 mg/mL, a dilution range of 0.15–1.00 mg/mL with five points, and an R^2^ value greater than 0.99, and total carotenoid content was calculated as the sum of individual compounds. Results were expressed as milligrams per 100 g of dry weight (mg/100 g DW).

#### 2.3.4. Phenol Profile

Phenolic compounds were quantified using the method described by Coyago et al. [22]. Twenty milligrams of freeze-dried sample were mixed with 500 μL of acidified methanol (80%), containing 0.1% HCl. The resulting solution was homogenised using a VM-300 vortex (Inter-biolab Inc., Orlando, FL, USA), followed by sonication in an FS60 ultrasonicator (Fisher Scientific Inc., Waltham, MA, USA). This procedure was repeated three times; after that, chromatographic analysis was carried out using an RRLC 1200 liquid chromatograph (Agilent Technologies, Mississauga, ON, Canada) equipped with a DAD-UV-VIS detector and a ZORBAX Eclipse Plus C18 column (5 μm, 4.6 mm, × 150 mm) (Agilent Scientific Instruments, Santa Clara, CA, USA). The mobile phase flow rate of 1 mL/min, considered a gradient of 0.01% formic acid solution (A) and acetonitrile HPLC grade (B), thus, 100% A, 0 min; 95% A + 5% B, 5 min; 50% A + 50% B, 20 min; and a 2 min column clean step. The injection volume was 20 µL, and detection was performed at 280 nm for flavanones and at 320 nm for hydroxycinnamic acids and flavones. Reference standards were used for identification and quantification, including shikimic acid, vanillic acid, *p*-hydroxybenzoic acid, 2-methoxybenzoic acid, 3-methoxybenzoic acid, 2,5-dihydroxybenzoic acid, gallic acid, *m*-coumaric acid, 3-hydroxybenzoic acid, protocatechuic acid, syringic acid, ellagic acid, tannic acid, *p*-coumaric acid, *o*-coumaric acid, chlorogenic, caffeic and ferulic, rutin, quercetin, myricetin, kaempferol, quercetin glucoside, chrysin, naringenin, naringin, luteolin, rutin, catechin, and epicatechin. Identification was performed by comparing the corresponding spectra and retention time. Quantitation was based on individual calibration curves with a concentration of 2 mg/mL, a dilution range of 0.3–2.00 mg/mL, with five points, and an R^2^ value greater than 0.99. The total phenolic content was calculated as the sum of all identified individual phenolics and expressed as milligrams of phenolic compound per 100 g of dry weight (mg/100 g dry weight).

### 2.4. Antioxidant Activity Analyses

Antioxidant activity by ABTS and DPPH was quantified using spectrophotometric methods [21]. Twenty milligrams of freeze-dried powder were mixed with 2 mL of methanol using a vortex mixer and shaken in an ultrasonic bath for 3 min. The supernatant was separated by microcentrifugation at 14,000 rpm for 5 min at 4 °C and stored under refrigeration until quantification.

The ABTS^•+^ radical cation was generated by mixing equal volumes of 7 mM ABTS (2,2′-azino-bis-(3-ethylbenzothiazoline-6-sulphonic acid)) with 2.45 mM potassium persulphate and allowing the reaction mixture to stand in the dark at room temperature for 16 h. The resulting ABTS^•⁺^ solution was subsequently diluted with absolute ethanol at a ratio of approximately 1:10, or until an absorbance of 0.70 ± 0.02 at 754 nm was achieved. For sample and standard analysis, 10 µL was pipetted into individual wells of a 96-well flat-bottom tissue culture plate (VWR, Novachen, Pittsburgh, PA, USA), followed by the addition of 200 µL of ABTS^•+^ radical. Absorbance was measured at 754 nm using a Themo Scientific Multiskan GO microplate reader (Agilent Scientific Instruments, Santa Clara, CA, USA).

The DPPH^•^ radical solution was prepared by dissolving 10 mg of DPPH in 50 mL of HPLC-grade methanol. For sample measurement, 20 µL of either standard or extract was added to 280 µL of DPPH^•^ radicals in each well of a 96-well flat-bottom tissue culture plate. In parallel, blanks were prepared by combining 300 µL of methanol and 300 µL of DPPH^•^ radicals. The plate was covered with aluminium foil to protect it from light and incubated on a 4310 Orbital Plate Shaker (Fisher Scientific, USA) for 30 min. Absorbance was measured at 560 nm using a BioTek microplate spectrophotometer (Agilent Scientific Instruments, Santa Clara, CA, USA). Antioxidant activity was quantified using a 2.5 nM Trolox standard, serially diluted (12.5%, 25%, 50%, and 75%) with an R^2^ greater than 0.99. The antioxidant capacity was expressed as millimoles of Trolox equivalents per 100 g of dry weight (mmol TE/100 g DW).

### 2.5. Antimicrobial Activity Analyses

#### 2.5.1. Antibacterial Activity

Antibacterial efficacy was evaluated using the well diffusion method according to the Clinical and Laboratory Standards Institute (CLSI) guidelines, with some modifications [22], against *Pseudomonas aeruginosa* ATCC 902, *Staphylococcus aureus* ATCC 6538P, *Escherichia coli* ATCC 8739, and *Streptococcus mutans* ATCC 25175. Microbial cultures were grown in brain heart infusion (BHI) broth, prepared in triplicate, and incubated aerobically at 37 °C for 48 h. Following incubation, the bacterial inoculum was adjusted to a turbidity equivalent to 1.5 × 10^8^ CFU/mL, corresponding to a 0.5 McFarland standard. These suspensions were spread onto Muller-Hinton agar plates. After that, using a micropipette tip, wells measuring 6 mm were made in the agar, and each well was filled with 80 µL of the test extract. The extract was made by mixing 2 g of freeze-dried powder with 10 mL of 50% ethanol, followed by sequential evaporation through lyophilisation. The solution for determining antibacterial activity was prepared by weighing 300 mg of the lyophilised dry extract and dissolving it in 1 mL of sterile distilled water. The mixture was vortexed until completely dissolved. The inoculated plates were incubated at 35 °C for 48 h. Streptomycin at a concentration of 1560 μg/mL was used as a positive control, whereas distilled water served as a negative control. Antimicrobial activity was determined by the well diffusion method, measuring the diameters of the inhibition zones formed around the wells, expressed in millimetres (mm).

Additionally, the antimicrobial activity tests were performed via the microdilution method. Bacterial strains were pre-cultured in brain heart infusion (BHI) overnight in a rotary shaker at 37 °C. Afterwards, each strain was adjusted to a final cell density. A stock solution of the tested compound was prepared by dissolving 400 mg of the lyophilised dry extract in 2 mL of sterile distilled water. Then, 180 μL of the solution was added to 20 μL of bacterial suspension, resulting in a final well volume of 200 μL in the microplate. The plates were incubated at 37 °C for 20 h. Microbial growth was evaluated by adding 20 μL of 2,3,5-triphenyltetrazolium chloride (TTC) at 37 °C for 2 h. This compound is reduced by metabolically active bacteria to form a red-coloured formazan product, allowing for easy visual detection of bacterial growth inhibition. The minimal inhibitory concentration (MIC) is defined as the lowest concentration of the antibacterial agent that completely inhibits the growth of the microorganism. These assays were performed at least in triplicate [23].

#### 2.5.2. Antifungal Activity

Antifungal activity was evaluated against *Candida tropicalis* (CC 13803) and *Candida albicans* (ATCC 1031). Yeast cultures were grown in yeast peptone dextrose broth, prepared in triplicate, and incubated aerobically at 30 °C for 48 h. Following incubation, the yeast inoculum was adjusted to a turbidity equivalent to 1.5 × 10^6^ CFU/mL, corresponding to a 0.5 McFarland standard. These suspensions were spread onto Sabouraud dextrose agar plates. After that, using a micropipette tip, wells measuring 6 mm were made in the agar, and each well was filled with 80 µL of the test extract. The extract was made by mixing 2 g of freeze-dried powder with 10 mL of 50% ethanol, followed by sequential evaporation through lyophilisation. The solution for determining antibacterial activity was prepared by weighing 300 mg of the lyophilised dry extract and mixing it with 1 mL of sterile distilled water. The inoculated plates were incubated at 35 °C for 48 h. Fluconazole (1250 µg/mL) was used as the positive control, while distilled water served as the negative control.

Additionally, antifungal activity was evaluated employing the microdilution technique. Fungal inocula were obtained from 24 h cultures grown in Yeast Peptone Dextrose Broth (YPDB) and adjusted to a turbidity equivalent to 0.5 MacFarland. A stock solution of the tested compound was prepared by dissolving 400 mg of the lyophilised dry extract in 2 mL of sterile distilled water. Aliquots of 180 μL of this solution were transferred into microplate wells containing 20 μL of fungal suspension, to a final volume of 200 μL per well. The microplates were incubated at 30 °C for 72 h. To assess metabolic activity, TTC (2,3,5-triphenyltetrazolium chloride) was added to each well; viable cells reduced TTC to a red colour, allowing visual determination of growth. The minimal inhibitory concentration (MIC) was defined as the lowest concentration of the extract that completely inhibited the fungal growth. All experiments were conducted in triplicate [23].

### 2.6. Statistical Analysis

The statistical analysis was conducted using STATGRAPHICS Centurion XVII (StatPoint Technologies Inc., Warrenton, VA, USA) and RStudio 4.3.3 (R Foundation for Statistical Computing, Vienna, Austria). All data were reported as means with standard deviation. Principal component analysis (PCA) was applied to identify the variables that contributed most significantly to the observed variability among the fruit samples. The PCA model encompasses all measured parameters, including carotenoids, phenolics, total organic acids, other bioactive constituents, antioxidant activity, and antimicrobial activity.

Since the variables were expressed in different units of measurement, data standardisation was performed before analysis. Each variable was centred by subtracting the mean and scaled by dividing by the standard deviation, thereby achieving a mean of zero and a variance of one. This normalisation procedure ensured that all variables contributed equally to the model, regardless of their original magnitude.

## 3. Results

### 3.1. Physicochemical Characteristics

Amazonian fruits have long been a staple of the traditional diet of indigenous communities. Table 1 shows the physicochemical characteristics of the fruits under study, with a focus on variables such as weight, size (longitudinal diameter and equatorial diameter), pH, total soluble solids, total titratable acidity, humidity, and ash content. Additionally, the concentrations of minerals such as calcium, iron, potassium, magnesium, and sodium are presented.

In this regard, considerable variability was observed in fruit weight, with values ranging from 0.2 g for *Miconia crenata* to 242.6 g for *Annona edulis*. The longitudinal diameter ranged from 9.2 mm for *M. crenata* to 112.7 mm for *Guatteria neuberthii*, and the equatorial diameter ranged from 7.3 mm for *M. crenata* to 80.8 mm for *A. edulis*. The pH values ranged from 4.3 (*Pourouma cecropiifolia*) to 6.1 (*A. edulis*). Soluble solids presented a wide range of values, from 1.0 °Brix (*A. edulis*) to 12.0 °Brix (*P. cecropiifolia*), while titratable acidity ranged from 0.1% (*Lacmellea oblongata*) to 4.4% (*M. crenata*). The humidity varied from 76.8% in *M. crenata* to 86.2% in *L. oblongata*.

The ash content ranged from 0.7% (*L. oblongata*) to 1.5% (*A. edulis*), which indicates the total mineral content. Regarding macro- and microminerals, the calcium content varied between 164.5 mg/100 g DW in *L. oblongata* and 1046.1 mg/100 g DW in *M. crenata*. Potassium was the most abundant mineral, with values ranging from 1111.1 mg/100 g DW in *L. oblongata* to 2422.7 mg/100 g DW in *P. cecropiifolia*, which reaffirms the importance of these fruits as sources of this nutrient. Magnesium ranged from 72.8 mg/100 g DW in *L. oblongata* to 131.1 mg/100 g DW in *M. crenata*, while sodium ranged from 13.8 mg/100 g DW in *M. crenata* to 67.4 mg/100 g DW in *L. oblongata*.

### 3.2. Analysis of Bioactive Compounds

The consumption of Amazonian fruits has provided indigenous communities with the nutrients and bioactive compounds necessary for health and well-being. Table 2 summarises the bioactive compounds found in the evaluated species, emphasising the concentration of vitamin C and organic acids. Additionally, the table includes profiles of carotenoids, chlorophylls, and their derivatives, as well as phenols, presenting the molecules with the highest concentrations.

The concentration of vitamin C varied significantly among the evaluated species, with values ranging from undetectable limits in *Lacmellea oblongata* to 25.4 mg/100 g of dry weight (DW) in *Grias neuberthii*. The range of citric acid was from 178.2 mg/100 g DW (*G. neuberthii*) to 3589.7 mg/100 g DW (*L. oblongata*), while the range of malic acid was from 26.6 mg/100 g DW (*P. cecropiifolia*) to 2703.4 mg/100 g DW (*G. neuberthii*). The range of tartaric acid was from 23.4 mg/100 g DW (*P. cecropiifolia*) to 901.3 mg/100 g DW (*A. edulis*). The total sum of organic acids ranged from 1063.6 mg/100 g DW (*P. cecropiifolia*) to 3887.7 mg/100 g DW (*L. oblongata*).

Molecules such as violaxanthin were identified in *G. neuberthii*; lutein with a range between 0.1 mg/100 g DW (*A. edulis*) and 4.1 mg/100 g DW (*M. crenata*); zeaxanthin with a value of 0.1 mg/100 g DW (*M. crenata* and *L. oblongata*); zeinoxanthin with a value of 0.2 mg/100 g DW (*M. crenata*); α-carotene with a value of 0.1 mg/100 g DW (*L. oblongata*) and 1.1 mg/100 g DW (*G. neuberthii*); and β-carotene with a value of 1.3 mg/100 g DW (*L. oblongata*) and 44.8 mg/100 g DW (*G. neuberthii*). The total carotenoid content as a sum of the major compounds ranged from 0.1 mg/100 g DW (*A. edulis*) to 46.1 mg/100 g DW (*G. neuberthii*).

The chlorophyll and derivative profile showed the presence of chlorophyll b and pheophytin b, with concentrations ranging from 0.2 mg/100 g DW in *A. edulis* to 27.2 mg/100 g DW in *M. crenata*, and from 9.5 mg/100 g DW in *P. cecropiifolia* to 17.8 mg/100 g DW in *M. crenata*. Additionally, the sum of the major chlorophyll and derivative compounds ranged from 0.2 mg/100 g DW (*A. edulis*) to 45.0 mg/100 g DW (*M. crenata*).

Gallic acid ranged from 4.7 mg/100 g DW in *M. crenata* to 403.2 mg/100 g DW in *A. edulis*. *A. edulis* contained 0.1 mg/100 g DW of catechin. Syringic acid ranged from 111.7 mg/100 g DW in *L. oblongata* and 955.3 mg/100 g DW in *M. crenata*; chlorogenic acid ranged from 213.8 mg/100 g DW (*L. oblongata*) to 1976.7 mg/100 g DW (*P. cecropiifolia*); and caffeic acid ranged from 1613.2 mg/100 g DW (*P. cecropiifolia*) to 3287.9 mg/100 g DW (*G. neuberthii*). *L. oblongata* showed the presence of naringenin (2086.1 mg/100 g DW). In comparison, ferulic acid was present at levels ranging from 311.9 mg/100 g DW (*G. neuberthii*) to 1445.5 mg/100 g DW in *P. cecropiifolia. G. neuberthii* presented ferulic acid (311.9 mg/100 g DW), kaempferol (15.6 mg/100 g DW), quercetin glycoside (24.3 mg/100 g DW), and quercetin (27.4 mg/100 g DW). Additionally, the total phenol content, calculated as the sum of the major individual compounds, ranged from 403.2 mg/100 g DW in *A. edulis* to 5064.3 mg/100 g DW in *P. cecropiifolia*.

### 3.3. Antioxidant Activity Analyses

The complex interaction between the different molecules present in plant matrices is closely related to the antioxidant capacity exhibited by these biological systems. This activity is primarily determined by the presence and synergy of phenolic compounds, flavonoids, carotenoids, and other secondary metabolites [24]. Table 3 presents the antioxidant activity of the extracts of the Amazonian fruits analysed, evaluated by the DPPH^•^ free radical and ABTS^•+^ radical cation inhibition assays, both widely used standardised methods to estimate the neutralisation capacity of reactive oxygen species.

Using the DPPH method, values ranged from 0.8 mmol TE/100 g DW for *A. edulis* to 4.2 mmol TE/100 g DW for *M. crenata*. Using the ABTS^•+^ method, values ranged from 2.6 mmol TE/100 g DW for *A. edulis* to 6.6 mmol TE/100 g DW for *L. oblongata*.

### 3.4. Antimicrobial Activity Analyses

The growing resistance of various microorganisms to traditional antimicrobial treatments has created a need to explore new sources of bioactivity. In this context, plant extracts are a promising alternative. Table 4 shows the antimicrobial activity of freeze-dried, 50% aqueous-ethanolic extracts obtained from Amazonian fruits. These extracts were tested against clinically relevant bacterial strains, including *Escherichia coli*, *Staphylococcus aureus*, *Pseudomonas aeruginosa*, and *Streptococcus mutans*. They were also tested against pathogenic yeasts in the *Candida* genus, specifically *C. albicans* and *C. tropicalis*. Additionally, Table 5 illustrates the minimum inhibitory concentrations of the fruits under investigation.

The antimicrobial activity against *E. coli* ranged from 8.0 mm (*G. neuberthii*) to 21.0 mm (*M. crenata*). Against *S. aureus*, the values ranged from 11.0 mm (*M. crenata*, *L. oblongata*, and *P. cecropiifolia*) to 14.0 mm (*G. neuberthii*); against *P. aeruginosa*, the values ranged from 8.5 mm in *P. cecropiifolia* to 14.2 mm in *M. crenata*; against *S. mutans*, values ranged from 10.0 mm in *M. crenata* and *G. neuberthii* to 12.0 mm in *L. oblongata* and *P. cecropiifolia*; and against *C. albicans* and *C. tropicalis*, values were 17.5 mm and 14.0 mm, respectively.

Evaluation of the minimum inhibitory concentration (MIC) revealed significant variability among species and microorganisms. Against *E. coli*, the values ranged from 2.7 mg/mL for *M. crenata* to 84.6 mg/mL for *G. neuberthii*, with *M. crenata* proving to be the most effective species against this bacterium. Against *P. aeruginosa*, values of 43.1 mg/mL were observed in *M. crenata*, compared to 85.9 mg/mL in *P. cecropiifolia*. For *S. aureus*, the values ranged from 10.6 mg/mL (*G. neuberthii*) to 86.3 mg/mL (*M. crenata*), and for *S. mutans*, they ranged from 10.4 mg/mL (*L. oblongata*) to 21.6 mg/mL (*M. crenata*). Finally, an MIC of 20.8 mg/mL was recorded in *L. oblongata* against both *C. albicans* and *C. tropicalis*.

### 3.5. Statistical Analysis

**Figure 2:**
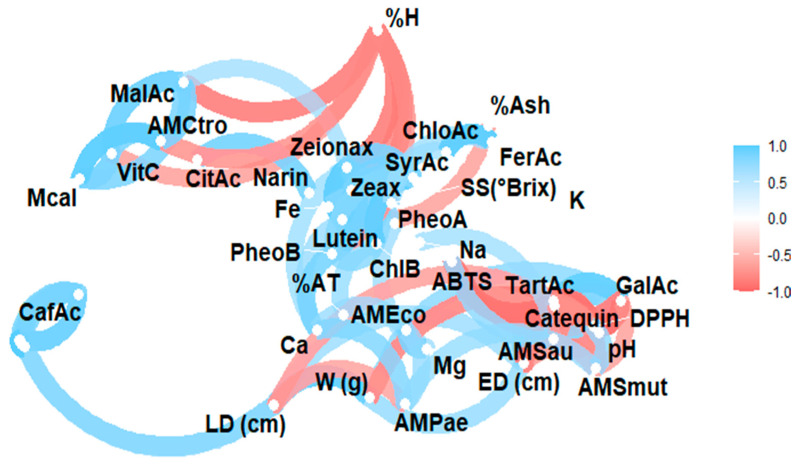
Pearson correlation analysis of the studied variables. Note: violaxanthin (Violax), lutein (Lutein), zeaxanthin (Zeax), zeionaxanthin (Zeioax), chlorophyll b (ChlB), pheophytin a (PheoA), pheophytin b (PheoB), chlorophyll a (ChlA), α-carotene (A-Car), β-carotene (B-Car), vitamin C (VitC), citric acid (CitAc), malic acid (MalAc), tartaric acid (TarAc), gallic acid (GalAc), syringic acid (SyrAc), chlorogenic acid (ChloAc), caffeic acid (CafAc), naringenin (Narin), ferulic acid (FerAc), kaempferol (Kamp), quercetin glycoside (QGlyc), quercetin (Quer), calcium (Ca), iron (Fe), potassium (K), magnesium (Mg), sodium (Na), antioxidant activity by ABTS (ABTS), antioxidant activity by DPPH (DPPH), antimicrobial activity against *S. aureus* (AMSau), antimicrobial activity against *E. coli* (AMEco), antimicrobial activity against *P. aeruginosa* (AMPae), antimicrobial activity against *C. albicans* (Mcal), antimicrobial activity against *C. tropicalis* (AMCtro).

**Figure 3:**
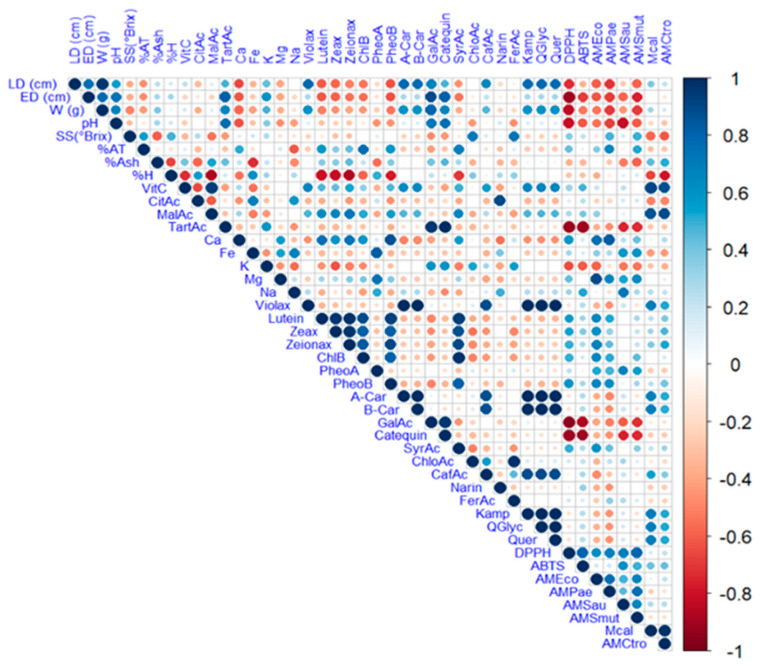
Heat map analysis of the studied variables. Note: violaxanthin (Violax), lutein (Lutein), zeaxanthin (Zeax), zeionaxanthin (Zeioax), chlorophyll b (ChlB), pheophytin a (PheoA), pheophytin b (PheoB), chlorophyll a (ChlA), α-carotene (A-Car), β-carotene (B-Car), vitamin C (VitC), citric acid (CitAc), malic acid (MalAc), tartaric acid (TarAc), gallic acid (GalAc), syringic acid (SyrAc), chlorogenic acid (ChloAc), caffeic acid (CafAc), naringenin (Narin), ferulic acid (FerAc), kaempferol (Kamp), quercetin glycoside (QGlyc), quercetin (Quer), calcium (Ca), iron (Fe), potassium (K), magnesium (Mg), sodium (Na), antioxidant activity by ABTS (ABTS), antioxidant activity by DPPH (DPPH), antimicrobial activity against *S. aureus* (AMSau), antimicrobial activity against *E. coli* (AMEco), antimicrobial activity against *P. aeruginosa* (AMPae), antimicrobial activity against *C. albicans* (MCal), antimicrobial activity against *C. tropicalis* (AMCtro).

The complex relationship between the various components of a plant matrix remains unknown. In this sense, Figure 2 illustrates the Pearson correlation among the study variables, including physicochemical characteristics, minerals, vitamin C, organic acids, carotenoids, chlorophylls and their derivatives, and phenols, as well as antioxidant and antimicrobial activity. Figure 3 displays a heat map, where blue indicates a positive correlation and red indicates a negative correlation.

**Figure 4:**
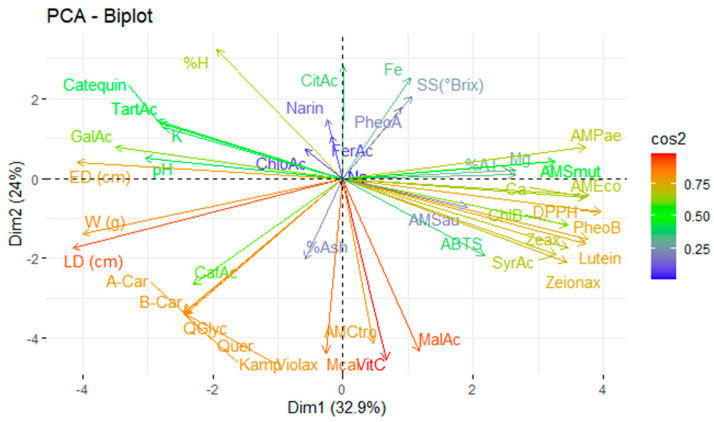
Principal component analysis of the studied variables. Note: violaxanthin (Violax), lutein (Lutein), zeaxanthin (Zeax), zeionaxanthin (Zeioax), chlorophyll b (ChlB), pheophytin a (PheoA), pheophytin b (PheoB), chlorophyll a (ChlA), α-carotene (A-Car), β-carotene (B-Car), vitamin C (VitC), citric acid (CitAc), malic acid (MalAc), tartaric acid (TarAc), gallic acid (GalAc), syringic acid (SyrAc), chlorogenic acid (ChloAc), caffeic acid (CafAc), naringenin (Narin), ferulic acid (FerAc), kaempferol (Kamp), quercetin glycoside (QGlyc), quercetin (Quer), calcium (Ca), iron (Fe), potassium (K), magnesium (Mg), sodium (Na), antioxidant activity by ABTS (ABTS), antioxidant activity by DPPH (DPPH), antimicrobial activity against *S. aureus* (AMSau), antimicrobial activity against *E. coli* (AMEco), antimicrobial activity against *P. aeruginosa* (AMPae), antimicrobial activity against *C. albicans* (Mcal), antimicrobial activity against *C. tropicalis* (AMCtro).

The complex interaction between the analysed variables, including physicochemical characteristics, mineral content, vitamin C, organic acids, carotenoids, chlorophylls and their derivatives, and phenolic compounds, as well as antioxidant and antimicrobial activity, requires multivariate statistical tools for proper interpretation. In this context, principal component analysis (PCA) reduces the dimensionality of the dataset by grouping the original variables into a smaller number of uncorrelated components that explain as much of the total variance as possible. Figure 4 illustrates the application of PCA to the variables under study, allowing patterns of association between bioactive compounds and their respective functional activities to be identified.

## 4. Discussion

### 4.1. Physicochemical Analyses

Amazonian fruits are a valuable resource for human health. Therefore, it is essential to analyse the fruits’ physical and chemical properties to determine their quality, which is crucial for consumer satisfaction and marketability. Additionally, analysing parameters such as pH, total soluble solids, and titratable acidity provides an indication of ripeness, which influences the fruit’s quality and potential shelf life [25].

Physicochemical results are partly consistent with those reported for tropical fruits, which can weigh anything from 0.4 ± 0.1 g (*Miconia crocea*) to 9117.2 ± 143.0 g (*Artocarpus heterophyllus*), have a longitudinal diameter ranging from 1.0 ± 0.2 cm (*M. crocea*) to 88.4 ± 7.2 cm (*Inga edulis*), and an equatorial diameter ranging from 0.7 ± 0.2 cm (*M. crocea*) to 27.5 ± 27.5 cm (*A. heterophyllus*) [6]. In the case of *M. crenata* (also known as *Clidemia hirta*), which is classified as an invasive species, the fruit is 6–8 mm long [26], consistent with the values found in this study. Conversely, the size of *G. neuberthii* was greater than the 400.3 ± 41.5 g reported by other authors, possibly due to the fruits being more advanced in their state of ripeness [17]. *A. edulis*, in turn, showed a larger size than that reported in other studies, ranging from 1.4 cm to 5.5 cm in length and from 1.0 cm to 4.0 cm in diameter [26], which could be attributed to genetic variations or agroecological conditions.

pH values are generally higher than those reported for tropical fruits, which range from 3.17 to 5.40 [27], and are within the range observed in a study of 51 tropical fruits, which ranged from 1.0 ± 0.0 (*M. alba*) to 8.2 ± 0.2 (*S. edulis*) [6]. Soluble solids and titratable acidity are crucial for determining the flavour and ripeness of the fruit. The high acidity of *M. crenata* may contribute to its acidic sensory profile. In contrast, the high soluble solids content of *P. cecropiifolia* suggests a fruit with a higher sugar content, consistent with its traditionally reported sweet flavour.

Humidity values are comparable to those found in *P. cecropiifolia* in other studies, which reported values of 84.5% and 82.9% + 0.6 [28,29]. However, higher humidity was reported in *G. neuberthii* than that published by other authors, who presented a value of 58.6% + 0.5 [17]. This difference could be due to the plants being harvested at a more mature stage, which naturally leads to a higher water content.

Lower ash values were observed in *G. neuberthii* compared to the 4.74% + 0.1 reported by other studies [17], which may reflect differences in the soil, climate, or genetics. The results obtained for *P. cecropiifolia* were consistent with those reported by other authors, who found values of 0.34% + 0.02 and 1.81% [28,29], confirming the variability of this parameter depending on the fruit’s origin.

Studies have shown that high calcium concentrations can inhibit iron absorption [30], which is consistent with the low iron values observed in most species, except *M. crenata* (12.6 mg/100 g DW) and *L. oblongata* (49.6 mg/100 g DW). In the case of *P. cecropiifolia*, iron levels were lower than those reported by other authors, who found a value of 3.9% [28]. However, its richness in phosphorus, potassium, and moderate amounts of calcium and sodium has been documented [26], which is consistent with the findings of this study.

### 4.2. Analysis of Bioactive Compounds

Studying the bioactive compounds in Amazonian fruits reveals their high nutritional and functional potential, despite many of these native species having been little explored scientifically. In this context, the antioxidant activity and health benefits of vitamin C, organic acids, carotenoids, chlorophylls, and phenolic compounds were analysed.

Ascorbic acid results demonstrate that *G. neuberthii* is an excellent source of this water-soluble antioxidant. In contrast, the vitamin C content of *P. cecropiifolia* was lower than that reported by other researchers (31%) [28], which could be due to differences in fruit maturity, analytical techniques, or growing conditions.

The main components in the organic acid profile were identified as citric, malic, and tartaric acids. Organic acid compounds play a crucial role in sensory perception (acidity), post-harvest preservation, and the bioavailability of minerals. Previous studies have shown that malic and citric acids are the primary contributors to the acidity of fruits such as *Litchi chinensis* [31], a finding consistent with the results obtained in this research.

The phenolic profile revealed the presence of gallic acid, catechin, syringic acid, chlorogenic acid, caffeic acid, naringenin, ferulic acid, kaempferol, quercetin glycoside, and quercetin. These results are consistent with previous studies reporting the presence of phenolic compounds in *G. neuberthii* [31] and *P. cecropiifolia* [28], as well as various Annona species [32]. Regarding *Miconia*, the literature describes a remarkable phytochemical richness, including flavonoids, triterpenes, steroids, phenolic acids, tannins, and aglycones such as quercetin, matteucinol, and kaempferol [33].

### 4.3. Antioxidant Activity Analyses

Evaluating the antioxidant activity of Amazonian fruits is essential for determining their functional potential and health benefits, particularly given their ability to neutralise free radicals involved in oxidative stress and chronic non-communicable diseases. This study quantified antioxidant activity using two widely validated methods: the inhibition of DPPH^•^ and ABTS^•+^ free radicals, expressed in mmol equivalents of Trolox per 100 g of dry weight (mmol TE/100 g DW).

Antioxidant activity by DPPH results highlights *M. crenata* as one of the species with the most significant capacity to neutralise free radicals in a lipophilic medium. This finding is consistent with previous studies that have documented the potent antioxidant activity of *Miconia* species [33]. This activity may be related to the abundance of phenolic compounds and flavonoids, such as quercetin and kaempferol, as well as phenolic acids, that have been identified in this species.

Antioxidant activity by ABTS results suggests that *L. oblongata* has a remarkable hydrophilic antioxidant capacity, potentially linked to its phenolic acid content, including chlorogenic, caffeic, and ferulic acids, as well as naringenin, a flavonoid with high radical-scavenging capacity. However, it is important to note that the ABTS values reported in this study were lower than those found by other researchers, who measured values of up to 119.0 mmol TE/kg in the mucilage of *Pourouma cecropiifolia* [29]. This discrepancy may be attributed to various factors, including the analysed tissue (pulp vs. mucilage), degree of ripeness, extraction method, and environmental and genetic variations between populations, as proposed by other researchers [34].

Overall, the results obtained here confirm that the evaluated Amazonian fruits are a relevant source of antioxidant compounds, albeit with significant variations between species. These differences are due to the intrinsic phytochemical diversity of each fruit and the polarity characteristics and mechanisms of action of each method: DPPH^•^ mainly detects lipophilic compounds, while ABTS^•+^ is sensitive to both hydrophilic and lipophilic compounds. Therefore, evaluating the antioxidant potential of these species using multiple methods provides a more comprehensive view, as suggested by other authors [6,23].

### 4.4. Antimicrobial Activity Analyses

The antimicrobial activity of Amazonian fruit extracts evaluated in this study reveals their potential as a natural source of bioactive compounds with therapeutic applications. Their effectiveness against Gram-positive bacteria (*Staphylococcus aureus* and *Streptococcus mutans*), Gram-negative bacteria (*Escherichia coli* and *Pseudomonas aeruginosa*), and pathogenic yeasts (*Candida albicans* and *Candida tropicalis*)—organisms commonly associated with clinical infections—was assessed.

Inhibitory activity against *E. coli*, *S. aureus*, and *S. mutans* was observed in all of the evaluated extracts, except for those from *Annona edulis*. This indicates that this species has low antimicrobial potential under the analysed conditions. By contrast, extracts from *Miconia crenata* and *Pourouma cecropiifolia* exhibited activity against *P. aeruginosa*, a bacterium renowned for its high antimicrobial resistance, underscoring the functional value of these species. Similarly, extracts from *Lacmellea oblongata* exhibited antifungal activity against *C. albicans* and *C. tropicalis*, suggesting their potential use as natural antifungal agents.

Minimum inhibitory concentration (MIC) results demonstrate differential microbial sensitivity profiles, depending on the type of extract, which may be influenced by the phytochemical composition, compound polarity, and mechanism of action. The observed efficiency in *M. crenata* is supported by reviews reporting antimicrobial activity in various *Miconia* species against bacteria and fungi, including *Escherichia coli*, *Staphylococcus aureus*, *Bacillus cereus*, *Vibrio cholerae*, *Salmonella choleraesuis*, *Klebsiella pneumoniae*, *Streptococcus pneumoniae*, *Candida krusei*, *Candida glabrata*, *S. aureus*, and *Pseudomonas aeruginosa* [33]. Similarly, studies on species of the genus Annona have demonstrated antibacterial and antifungal activity against *S. aureus*, *E. coli*, *P. aeruginosa*, *C. albicans*, and *C. tropicalis*. This is partially consistent with the results observed in *A. edulis*, although no significant activity was detected in this case [32].

The absence of an antimicrobial effect in *A. edulis* may be due to the extract evaluated having a low concentration of, or an absence of, active antimicrobial compounds, or to the extract having a predominance of low-molecular-weight compounds that are ineffective against the tested microorganisms. In contrast, the high effectiveness of *M. crenata* may be related to the presence of previously documented flavonoids and phenolic acids, which act on the microbial cell membrane by interfering with protein synthesis or generating lethal oxidative stress [33,35].

### 4.5. Statistical Analysis

Correlation analysis of the physicochemical, functional, antioxidant, and antimicrobial properties of Amazonian fruits revealed significant relationships that help us to understand the metabolic interactions between these compounds and their impact on the fruits’ functional and microbiological quality. A direct relationship was observed between weight and size, consistent with developmental physiology, where an increase in plant biomass is accompanied by proportional expansion of cells in the longitudinal and equatorial dimensions, as suggested by other authors [36,37]. Similarly, a correlation was detected between malic acid and vitamin C, supporting previous studies that have shown malic acid contributes to maintaining the reduced state of ascorbic acid by promoting functional stability through the action of malate dehydrogenase. This favours the formation of NADPH and thus the regeneration of ascorbic acid [38].

Tartaric acid was positively correlated with equatorial diameter, weight, and pH. This finding is consistent with previous studies on grapes, which have shown that this organic acid accumulates in medium-sized berries, suggesting an optimal range for its biosynthesis [39]. These associations may reflect the fruit’s metabolic adaptation to specific growth and ripening conditions.

In terms of minerals, iron was found to be correlated with moisture and citric acid, while magnesium was found to be associated with calcium and iron. Sodium, meanwhile, was found to be associated with citric acid and iron. These interactions reflect a mineral balance influenced by the availability of bivalent cations and organic acids, which act as chelating agents. Furthermore, as documented, high calcium concentrations can inhibit iron absorption, and low magnesium levels can reduce bioavailability [30]. Conversely, malic acid has been reported to promote calcium accumulation in fruits such as *Cerasus humilis* [40].

In the case of carotenoids, positive correlations were found between violaxanthin and variables such as weight and length, as well as vitamin C and malic acid. Similarly, positive correlations were found between lutein, titratable acidity, malic acid, and calcium. The intercorrelation between carotenoids (zeaxanthin, zeinoxanthin, α-carotene, and β-carotene) indicates common regulation of the biosynthetic pathway, whereby the enzymes involved in converting precursors are subject to shared mechanisms, as detailed in prior studies [41]. Additionally, larger fruits, such as persimmons, tend to exhibit greater colour intensity, which is correlated with high carotenoid levels; however, this may be offset by the dilution of specific mineral nutrients [42].

Chlorophylls and pheophytins also exhibited significant correlations with mineral and pigment compounds, suggesting their potential co-accumulation in active photosynthetic tissues and their association with the oxidative degradation processes related to maturation and senescence [43].

Regarding phenolic compounds, an association was found between gallic acid and morphological parameters (e.g., diameter, weight, and pH), as well as between catechin and gallic acid. This indicates a possible biosynthetic synergy. Syringic acid, chlorogenic acid, caffeic acid, and ferulic acid were found to be correlated with minerals, soluble solids, and carotenoids, which confirms the complexity of metabolic regulation. These associations reflect the interconnected nature of the biosynthetic pathways involved, which include enzymes such as phenylalanine ammonium lyase, chalcone synthase, and specific transferases [44].

Conversely, compounds such as kaempferol, quercetin glycoside, and free quercetin were closely related to morphological parameters, vitamin C, carotenoid pigments, and caffeic acid. These correlations suggest a pattern of co-accumulation in response to natural oxidative stress in fruits, particularly in external or exposed tissues [41].

Finally, relevant associations were observed between bioactive compounds and antimicrobial activity. Activity against *E. coli* was found to be associated with minerals (calcium and magnesium), pigments (lutein, zeaxanthin, and chlorophyll b), pheophytin a, and phenolic compounds (syringic acid), as well as with DPPH antioxidant capacity. This indicates that certain antioxidant compounds may exhibit synergistic antimicrobial properties, as has been documented for flavonoids and chlorogenic acid in the presence of minerals that influence pH levels or ion exchange [35].

Similarly, activity against *P. aeruginosa* and *S. aureus* was correlated with antioxidant variables, minerals, and pigments. This indicates a complex interaction between the medium’s oxidative capacity, phenolic compounds, and microbial resistance. The most extensive correlations were observed in S. mutans, reflecting this bacterium’s sensitivity to multiple bioactive factors.

Regarding fungi, activity against *C. albicans* and *C. tropicalis* exhibited a strong correlation with vitamin C, malic acid, carotenoids (violaxanthin, α-carotene, and β-carotene), and phenolic compounds (e.g., caffeic acid, kaempferol, quercetin glucoside, and quercetin). These results reinforce the hypothesis that specific combinations of antioxidants and phenols can modulate fungal activity, with potential applications in natural antifungal products [45].

Correlation analysis showed an inverse relationship between moisture and ash; vitamin C and moisture; citric acid with ash and vitamin C; malic acid with moisture and citric acid; calcium with longitudinal diameter, equatorial diameter, weight, pH, moisture and citric acid; iron with ash and malic acid; sodium with titratable acidity, ash, and potassium; lutein, zeaxanthin, zeinoxanthin, chlorophyll b with longitudinal diameter, equatorial diameter, weight, moisture content, and potassium; pheophytin b with longitudinal diameter, equatorial diameter, weight, pH, and moisture content; syringic acid with moisture content; DPPH, ABTS, antimicrobial activity against *E. coli*, *P. aeruginosa*, *S. aureus*, and *S. mutans* with equatorial diameter, longitudinal diameter, weight, pH, tartaric acid, potassium, gallic acid, and catechin; and antimicrobial activity against *C. albicans* and *C. tropicalis* with soluble solids, moisture, and citric acid. A negative correlation was observed between citric acid and soluble solids content [40].

The analysis of inverse correlations revealed significant associations between physicochemical, nutritional, pigmentary, and functional variables, shedding light on the metabolic interactions that occur during the development, ripening, and bioactive expression of Amazonian fruits. An inverse relationship was identified between moisture and ash, indicating that an increase in water content leads to a decrease in mineral concentration on a dry basis. This phenomenon was also evident in the inverse relationship between vitamin C and moisture content, consistent with reports from other studies, where high water content and early ripening can dilute water-soluble antioxidant compounds [46].

Similarly, citric acid showed a negative correlation with ash and vitamin C, suggesting a possible antagonism between organic acidity and mineral accumulation, which affects the stability of ascorbic acid. Similarly, malic acid was found to correlate negatively with both moisture and citric acid, suggesting that these acids are oppositely regulated during fruit ripening.

There was an inverse relationship between calcium and multiple morphological and physicochemical variables (longitudinal diameter, equatorial diameter, weight, pH, moisture, and citric acid). This reflects a possible dilution effect of the mineral in larger fruits with a higher water content, as well as an acid–mineral balance that affects the accumulation of this nutrient. These observations are consistent with studies showing a decrease in calcium in larger fruits [42].

Negative correlations were also found between iron, ash, and malic acid, which can be explained by interference with iron absorption in the presence of other minerals, such as calcium and magnesium, or organic acids that form non-bioavailable complexes [30].

Sodium exhibited an inverse relationship with titratable acidity, ash, and potassium, reflecting ionic competition between cations in the osmotic and nutritional balance of the fruit. This type of interaction has been documented as part of the way fruits adjust to water and salt stress conditions in terms of their mineral composition.

About pigments, a negative correlation was observed between lutein, zeaxanthin, zeinoxanthin, and chlorophyll b on the one hand, and fruit size, moisture content, and potassium on the other hand. This could indicate a higher concentration of these pigments in smaller, less watery fruits, possibly as an adaptive mechanism in response to greater light exposure or oxidative stress.

Pheophytin b also showed an inverse relationship with morphological and pH variables, which may reflect the advanced degradation of chlorophyll during senescence or storage. Syringic acid was negatively correlated with moisture content, suggesting a preferential accumulation in more concentrated or mature tissues.

Notably, antioxidant (DPPH and ABTS) and antimicrobial activities against *E. coli*, *P. aeruginosa*, *S. aureus*, and *S. mutans* exhibited negative correlations with morphological and water content variables. This suggests that smaller, denser fruits with higher phenolic content may exhibit greater bioactivity.

Conversely, antimicrobial activity against *C. albicans* and *C. tropicalis* was found to be negatively correlated with soluble solids, moisture, and citric acid. This finding supports the hypothesis that these fungi may be more susceptible to environments with lower moisture content and lower acidity. Previous studies have indeed documented a negative correlation between citric acid and soluble solids, reflecting the natural antagonism between sour and sweet flavours in fruits during ripening [47].

Principal component analysis explained 56.9% of the total variability in the system, distributed between the first principal component (32.9%) and the second principal component (24.0%). This indicates that a robust dimensional reduction model exists, capable of identifying the variables with the most significant explanatory power in distinguishing between the studied fruits.

Antioxidant activity (as measured by the ABTS and DPPH assays) was found to be strongly influenced by pigments such as pheophytin b, lutein, zeaxanthin, and zeinoxanthin. This highlights the direct link between antioxidant bioactivity and carotenoid content in these fruits, consistent with their ability to neutralise free radicals in polar and non-polar media [23].

Similarly, the antimicrobial activity against *E. coli* was related to the calcium content and the DPPH antioxidant activity. This suggests a possible synergistic effect between the antioxidant capacity and the microbial bioactivity. In turn, antimicrobial activity against *P. aeruginosa* was attributed to the magnesium content, while activity against pathogenic yeasts (*C. albicans* and *C. tropicalis*) was primarily influenced by vitamin C. This supports its recognised role as an antimicrobial agent through the direct or indirect oxidation of cell structures [48].

The most decisive variables in the PCA model were fruit size (longitudinal and equatorial diameter) and bioactive compounds, including quercetin, β-carotene, α-carotene, kaempferol, quercetin glycoside, violaxanthin, vitamin C, and malic acid. These compounds are recognised for their antioxidant, antimicrobial, and nutraceutical effects.

The relevance of the identified bioactive compounds, such as phenolic acids (e.g., chlorogenic, caffeic, ferulic, syringic), flavonoids (kaempferol, quercetin, and their glycosides), carotenoids (β-carotene, lutein, violaxanthin), and vitamin C, is that they are molecules with potential for the development of new products. These compounds are known for their antioxidant, anti-inflammatory, antimicrobial, and skin-protective properties, which support their use in functional food formulations (e.g., beverages, nutraceutical powders, natural preservatives), cosmetic products (e.g., anti-ageing creams, antioxidant serums), and pharmaceutical preparations (e.g., antimicrobial agents, supplements for oxidative stress-related conditions).

In this context, despite the promising results, this study has certain limitations. The research was limited to a single sampling period and a specific geographic region within the Ecuadorian Amazon, so it may not capture the full seasonal or ecological variability of the species studied. Although the study employed robust analytical techniques to quantify bioactive compounds and assess functional properties, the evaluation was conducted under in vitro conditions. Therefore, the bioavailability, metabolism, and synergistic interactions of these compounds in vivo remain to be investigated. Furthermore, antimicrobial activity was evaluated against selected standard microbial strains. Future studies should incorporate clinical isolates to reflect pathogenic diversity and resistance profiles more accurately. Finally, while the study highlights correlations between bioactive compounds and biological activity, the causal mechanisms remain unclear and require further biochemical and molecular validation.

## 5. Conclusions

The Ecuadorian Amazon is an invaluable source of nutritional biodiversity for local communities, offering fruits with functional properties and potential health benefits. Among the species evaluated, *Miconia crenata* exhibited the highest levels of total titratable acidity, calcium, magnesium, lutein, zeinoxanthin, chlorophyll b, pheophytin b, and syringic acid. It also showed potent antioxidant activity (DPPH assay) and a low minimum inhibitory concentration against *Escherichia coli*. *Grias neuberthii* stood out due to its high content of malic acid, violaxanthin, α-carotene, β-carotene, caffeic acid, kaempferol, and quercetin glycoside, and demonstrated notable inhibitory activity against *Staphylococcus aureus*. *Lactuca oblongata* was rich in iron, sodium, citric acid, and naringenin, and exhibited potent antioxidant activity (as measured by the ABTS assay) and antimicrobial activity against *Streptococcus mutans*, *Candida albicans*, and *Candida tropicalis*. *Pourouma cecropiifolia* was characterised by high concentrations of soluble solids, chlorogenic acid, and ferulic acid, while Annona edulis exhibited high levels of pH, tartaric acid, gallic acid, and catechin. Multivariate analyses, including correlation and principal component analyses, confirmed that fruit size, vitamin C content, carotenoid content (including lutein, β-carotene, and violaxanthin), flavonoid content (including kaempferol and quercetin derivatives), and mineral content (calcium and magnesium) significantly influence the functional activity of the extracts. Overall, these results underscore the nutritional and nutraceutical value of Amazonian fruits, as well as their potential as a source of natural ingredients with antioxidant and antimicrobial properties for the food, pharmaceutical, and cosmetic industries.

## Figures and Tables

**Table 1:** Average values of the physicochemical characteristics of Amazonian fruits.

	*Miconia crenata*	*Grias neuberthii*	*Lacmellea oblongata*	*Pourouma cecropiifolia*	*Annona edulis*
Weight (g)	0.2 ± 0.0	229.9 ± 7.8	5.9 ± 1.5	4.4 ± 0.6	242.6 ± 0.1
Longitudinal diameter (mm)	9.2 ± 0.4	112.7 ± 11.4	21.1 ± 8.2	22.4 ± 1.2	83.6 ± 0.3
Equatorial diameter (mm)	7.3 ± 0.1	47.1 ± 2.7	23.3 ± 1.8	20.9 ± 1.5	80.8 ± 0.1
pH	5.1 ± 0.0	5.0 ± 0.0	5.1 ± 0.0	4.3 ± 0.0	6.1 ± 0.0
Total soluble solids (°Brix)	8.0 ± 0.0	3.0 ± 0.0	5.7 ± 0.6	12.0 ± 1.0	1.0 ± 0.0
Total titratable acidity (%)	4.4 ± 0.5	0.2 ± 0.0	0.1 ± 0.0	2.0 ± 0.2	0.2 ± 0.0
Humidity (%)	76.8 ± 3.2	80.0 ± 0.9	86.2 ± 0.9	84.2 ± 0.7	83.2 ± 0.8
Ash (%)	0.9 ± 0.3	1.1 ± 0.0	0.7 ± 0.1	0.9 ± 0.1	1.5 ± 0.5
**Mineral profile (mg/100 g DW)**					
Ca	1046.1 ± 34.6	258.2 ± 13.8	164.5 ± 9.5	998.1 ± 57.6	430.1 ± 4.6
Fe	12.6 ± 1.2	nd	49.6 ± 0.0	nd	nd
K	1373.3 ± 74.4	1643.9 ± 38.7	1111.1 ± 0.1	2422.7 ± 11.2	2389.1 ± 11.5
Mg	131.1 ± 21.4	87.3 ± 0.5	75.8 ± 0.5	72.8 ± 9.6	91.4 ± 4.6
Na	13.8 ± 1.9	55.7 ± 0.0	67.4 ± 2.4	27.5 ± 3.3	17.6 ± 1.4

Note: nd, Undetectable limit.

**Table 2:** Average values of the bioactive compounds of Amazonian fruits.

		*Miconia crenata*	*Grias neuberthii*	*Lacmellea oblongata*	*Pourouma cecropiifolia*	*Annona edulis*
Ascorbic acid (mg/100 g DW)		12.5 ± 0.3	25.4 ± 0.7	nd	6.6 ± 0.2	0.2 ± 0.0
Organic acid profile (mg/100 g DW)						
	Citric acid	379.8 ± 47.6	178.2 ± 19.8	3589.7 ± 11.4	1013.6 ± 1.9	583.8 ± 4.9
	Malic acid	1845.5 ± 29.8	2703.4 ± 63.5	255.2 ± 18.1	26.6 ± 4.6	350.0 ± 4.3
	Tartaric acid	40.6 ± 2.2	37.6 ± 2.9	42.9 ± 3.1	23.4 ± 0.7	901.3 ± 20.8
	Total organic acid	2265.9 ± 34.4	2919.2 ± 86.2	3887.7 ± 13.6	1063.6 ± 7.2	1835.2 ± 21.4
Carotenoid profile (mg/100 g DW)						
	Violaxanthin		0.1 ± 0.0			
	Lutein	4.1 ± 0.6		0.6 ± 0.0	1.0 ± 0.0	0.1 ± 0.0
	Zeaxanthin	0.1 ± 0.0		0.1 ± 0.0		
	Zeionaxanthin	0.2 ± 0.0				
	α-carotene		1.1 ± 0.0	0.1 ± 0.0		
	β-carotene		44.8 ± 0.8	1.3 ± 0.0		
	Total carotenoid	4.4 ± 0.7	46.1 ± 0.8	4.8 ± 0.0	10.5 ± 1.1	0.1 ± 0.0
Chlorophylls and their derivatives (mg/100 g DW)						
	Chlorophyll b	27.2 ± 2.9		2.8 ± 0.0		0.2 ± 0.0
	Pheophytin b	17.8 ± 4.3			9.5 ± 0.9	
	Total chlorophylls	45.0 ± 2.7		2.8 ± 0.0	9.5 ± 0.9	0.2 ± 0.0
Total phenols (mg/100 g DW)						
	Galic acid	4.7 ± 0.1	124.2 ± 3.6	11.4 ± 0.5	28.8 ± 1.8	403.2 ± 3.2
	Cathechin					0.1 ± 0.0
	Syringic acid	955.3 ± 29.2	124.3 ± 6.5	111.7 ± 0.5		
	Chlorogenic acid		734.2 ± 35.7	213.8 ± 16.9	1976.7 ± 19.2	
	Caffeic acid		3287.9 ± 24.4		1613.2 ± 0.2	
	Naringenin			2086.1 ± 32.6		
	Ferulic acid		311.9 ± 12.6		1445.5 ±53.5	
	Kamferol		15.6 ± 0.4			
	Quercetin glycoside		24.3 ± 0.8			
	Quercetin		27.4 ± 0.9			
	Total phenols	960.0 ± 29.4	4650.0 ± 29.7	2423.0 ± 48.5	5064.3 ± 13.6	403.2 ± 3.2

Note: nd, Undetectable limit.

**Table 3:** Average values of the antioxidant activity of Amazonian fruits.

		*Miconia crenata*	*Grias neuberthii*	*Lacmellea oblongata*	*Pourouma cecropiifolia*	*Annona edulis*
**Antioxidant Activity (mmol TE/100 g DW)**						
	DPPH	4.2 ± 0.3	2.9 ± 0.2	3.6 ± 0.1	3.7 ± 0.1	0.8 ± 0.1
	ABTS	5.5 ± 0.9	6.4 ± 0.1	6.6 ± 0.1	5.9 ± 0.4	2.6 ± 0.1

**Table 4:** Average values of the antimicrobial activity of Amazonian fruits.

	Zone of Inhibition (mm)					
	*Bacterial strain*				*Fungal strain*	
Fruit Extracts	*E. coli* ATCC 8739	*S. aureus* ATCC 6538P	*P. aeruginosa* ATCC 9027	*S. mutans* ATCC 25175	*C. albicans* ATCC 1031	*C. tropicalis* ATCC 13803
*Miconia crenata*	21.0 ± 1.0	11.0 ± 0.5	14.2 ± 1.0	10.0 ± 0.5		
*Grias neuberthii*	8.0 ± 0.5	14.0 ± 1.2	-	10.0 ± 1.2	-	-
*Lacmellea oblongata*	16.0 ± 1.0	11.0 ± 0.0	-	12.0 ± 1.2	17.5 ± 0.6	14.0 ± 1.2
*Pourouma cecropiifolia*	14.0 ± 1.0	11.0 ± 0.5	8.5 ± 0.6	12.0 ± 2.3	-	-
*Annona edulis*	-	-	-	-		
Control	24.5 ± 0.5	28.0 ± 1.0	25.0 ± 1.2	28.0 ± 0.5	12.0 ± 0.5	17.0 ± 1.0

Note: non-active at the tested concentrations.

**Table 5:** Minimal inhibitory concentration of Amazonian fruits.

Microbial Strain	Minimal Inhibitory Concentration (mg/mL)				
	*Miconia crenata*	*Grias neuberthii*	*Lacmellea oblongata*	*Pourouma cecropiifolia*	*Annona edulis*
*E. coli* ATCC 8739	2.7	84.6	31.3	10.7	-
*P. aeruginosa* ATCC 9027	43.1	-	-	85.9	-
*S. aureus* ATCC 6538P	86.3	10.6	83.3	85.9	-
*S. mutans* ATCC 25175	21.6	21.1	10.4	21.5	-
*C. albicans* ATCC 1031	-	-	20.8	-	
*C. tropicalis* ATCC 13803	-	-	20.8	-	

Note: non-active at the tested concentrations.

## Data Availability

The original contributions presented in this study are included in the article. Further inquiries can be directed to the corresponding author.
